# Gastroesophageal junction pancreatic heterotopia/metaplasia: a case series

**DOI:** 10.1093/jscr/rjaf390

**Published:** 2025-06-09

**Authors:** Moaaz Amir, Salwa Sheikh

**Affiliations:** Imam Abdulrahman Bin Faisal University, College of Medicine, King Faisal Ibn Abd Al Aziz, King Faysal University, 34212, Eastern Province, Dammam, Saudi Arabia; Johns Hopkins Aramco Healthcare, Department of Pathology, 8131 Medical Access Rd No. 1, 34465, Eastern Province, Dhahran, Saudi Arabia

**Keywords:** gastroesophageal, pancreas, heterotopia, GEJ

## Abstract

Gastroesophageal junction pancreatic heterotopia is an uncommon congenital condition where pancreatic tissue is present outside its normal anatomical location. We present a case series of four patients who have presented with nonspecific, vague gastrointestinal symptoms that included abdominal pain, dyspepsia, and heartburn. Endoscopy was performed and revealed irregular Z-lines in three out of the four patients, with high suspicion of Barrett’s esophagus. Further histopathologic evaluation confirmed ectopic pancreatic tissue in all four patients, two of whom had mild chronic inflammation, another presented with chronic gastritis as well as reflux esophagitis, and the last patient exhibited intestinal metaplasia. Also, three patients were treated with proton pump inhibitors and were kept under constant surveillance, while one patient was lost to follow-up. Pancreatic heterotopia involving the gastroesophageal junction is a rare presentation that usually goes unnoticed due to its asymptomatic nature. It is important that clinicians recognize such presentations to provide optimal treatment.

## Introduction

Ectopic or heterotopic pancreas is a congenital anomaly with pancreatic tissue present outside of its normal location, with some authorities believing it is a metaplastic event. It is often asymptomatic and discovered incidentally. The most common location includes stomach, followed by duodenum, jejunum, colon, Meckel's diverticulum, and esophagus. Heterotopic pancreas involving gastroesophageal junction (GEJ) is extremely rare.

## Case series

**Figure 1:**
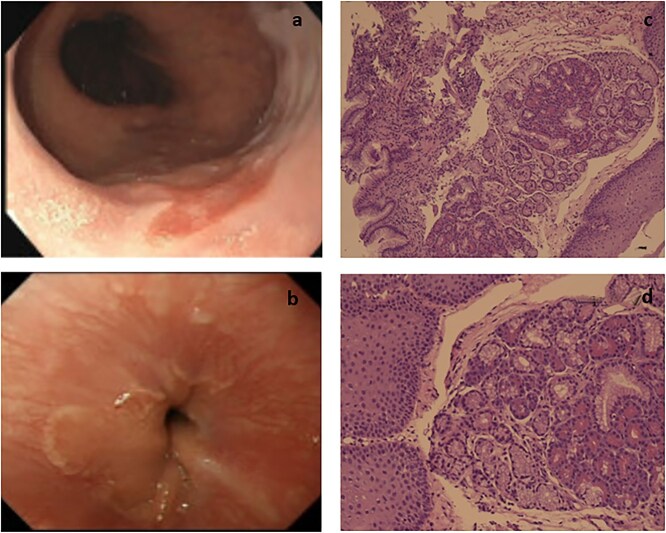
Endoscopic and pathological images. (a) GEJ showing irregular salmon colored patch. (b) Distal esophagus exhibiting a patch with irregular borders. (c) Section shows pancreatic lobules with esophageal squamous mucosa and gastric type mucosa of the GEJ (10×). (d) Higher power showing the squamous mucosa of GEJ with underlying pancreatic tissue (40×).

We herein report four cases of ectopic pancreatic tissue involving GEJ. The patients presented with nonspecific symptoms of abdominal pain, heartburn, dyspepsia, and vomiting. On endoscopy, three patients showed irregular Z-line with salmon-colored patch, and the clinical impression was to rule out Barrett's esophagus. One patient was referred from an outside hospital and had no in-house endoscopy available for review. All patients were biopsied and on histopathologic examination, showed heterotopic pancreatic tissue: two cases had additional mild chronic inflammation, one case with associated reflux esophagitis and moderate chronic gastritis, and one patient exhibited intestinal metaplasia in addition to the pancreatic heterotopia (Table 1) (Fig. 1). Three patients were given proton pump inhibitors (PPIs) and were put under surveillance; no further subsequent management was necessary. The patient referred from outside was lost for follow-up.

**Table 1:** Overview of GEJ pancreatic heterotopia/metaplasia cases reported in the case series

Case	Age	Sex	Presentation	Endoscopic finding	Management	Pathology	Follow up
1	45	F	Abdominal pain R/O GE reflux	Irregular Z-line with 2 cm long tongue-like projection at the GEJ, R/O Barrett’s esophagus	Biopsy with no subsequent further surgical intervention	Pancreatic acinar metaplasia/heterotopia and mild chronic inflammation	PPI and surveillance
2	41	F	Dyspepsia and vomiting	Hiatal hernia and short tongue of salmon colored mucosa at GE junction, R/O Barrett’s esophagus	Biopsy with no subsequent further surgical intervention	Pancreatic acinar metaplasia/heterotopia and mild chronic inflammation	PPI, dietary modification and surveillance
3	31	F	Abdominal pain R/O GE reflux	Small salmon colored mucosa at GE junction	Biopsy with no subsequent further surgical intervention	Pancreatic acinar metaplasia/heterotopia, reflux esophagitis, and moderate chronic gastritis	PPI, and surveillance (Patient was taking NSAID for sacroiliitis)
4	50	F	Outside diagnosis: Esophagitis R/O Barrett’s	No endoscopy report as case was referred from outside institution	Biopsy with no subsequent further surgical intervention	Pancreatic acinar metaplasia/heterotopia, and focal intestinal metaplasia	Lost for follow up

## Discussion

Ectopic pancreas/heterotopia is referred to as pancreatic tissue present in a location not connected to the pancreas or its ductal system. It is mostly considered a congenital anomaly; however, some believe it could be a metaplastic change. The most common location of pancreatic heterotopia is the upper gastrointestinal tract, specifically stomach, usually involving the greater curvature and 5 cm from the pylorus, followed by duodenum, and proximal jejunum [1–4]. Involvement of GEJ and esophagus is extremely rare [3, 5–8]. To date, <20 cases of heterotopic pancreas in the esophagus and <5 cases involving the hiatal hernia have been reported in literature [9]. It is reported to be predominant in men than women and is typically reported in 5th to 6th decade, with only a few cases reported in children [4].

Usually it is asymptomatic; however, symptoms vary depending on the location and size of the lesion, and any associated pathological condition, such as inflammation, obstruction of ducts, and extremely rare occurrence of malignant transformation [3]. In such cases, patients often present with abdominal pain, nausea, dysphagia, dyspepsia, heartburn, and rarely bleeding. Endoscopically, the characteristic findings are a submucosal nodule covered by normal mucosa. Some cases may show central umbilication. The lesions are often misdiagnosed as gastrointestinal stromal tumors [3].

The definitive diagnosis is made only by histopathologic examination with identification of peripancreatic tissue in this ectopic location. Microscopically, ectopic pancreas is classified into 4 types, originally described by Heinrich in 1909 as 3 types and sequentially modified in 1973 by Gasper-Fuentes [10]: Type 1 contains all elements of normal pancreatic tissue (acini, ducts, and islets), Type 2 contains acini and ducts (no islets) in Heinrich classification and Ducts only in Gasper-Fuentes classification, Type 3 contains ducts only in Heinrich classification and acini only in Gasper-Fuentes classification, and the extremely rare Type 4 of Gasper-Fuentes classification, which does not exist in Heinrich classification, and contains Islet cells only [11].

Treatment depends on symptoms and location; however, most patients are managed conservatively with observation/surveillance, and medical treatment commonly with PPIs, with surgical resection used in any rare cases.

## Conclusion

Awareness of this entity is extremely important since nonspecific symptoms of dyspepsia, abdominal pain, and heartburn may lead to incorrect clinical diagnosis if not continued by biopsy, and therefore will lead to no response to therapy.

## Data Availability

The data used to support the findings of this study are included within the article.
